# The E3 ligase MEX3B forms a tripartite complex with *Rest* and *Hotair* to determine the proliferative capacity of neural progenitor cells

**DOI:** 10.1098/rsob.250164

**Published:** 2025-09-10

**Authors:** Kamakshi Garg, Gourav Sharma, Sarbani Samaddar, Sourav Banerjee

**Affiliations:** ^1^ National Brain Research Centre, Manesar, Haryana, India

**Keywords:** long non-coding RNA, REST, neural progenitor cells, RNA-binding ubiquitin ligase, MEX3B

## Abstract

E3 ubiquitin ligases regulate the cellular proteome *via* proteasome-dependent protein degradation; however, there exist limited studies outlining their non-canonical functions. RNA-binding ubiquitin ligases (RBULs) represent a subset of E3 ligases that harbour RNA-binding domains, making them uniquely positioned to function as both RNA-binding proteins and E3 ligases. Our initial microarray screen for E3 ligases from mouse cortical neural progenitor cells identified MEX3B, a known RNA-binding ubiquitin ligase, to be differentially expressed. Here, we characterize the non-canonical role of MEX3B in the context of neural proliferation. We find that MEX3B is significantly reduced following the differentiation of neural progenitor cells (NPCs). The knockdown of MEX3B blocks the proliferative state of NPCs and leads to the enhancement of neurite length and dendrite branching. We observed that MEX3B regulates the stability of *Rest* mRNA in proliferative NPCs. Mechanistically, MEX3B interacts with *Rest* mRNA and the lncRNA *Hotair* to form a tripartite complex in the presence of basic fibroblast growth factor (bFGF). Loss of *Hotair* disrupts this complex; conversely, MEX3B RNAi significantly reduces *Hotair* abundance. *Rest* mRNA levels remain unaffected by *Hotair* knockdown, suggesting that the latter acts as a scaffold to facilitate bFGF-dependent MEX3B*–Rest* interaction in the MEX3B*–Rest–Hotair* tripartite axis. Our study demonstrates an RNA-driven post-transcriptional mechanism underlying NPC proliferation.

**Figure:**
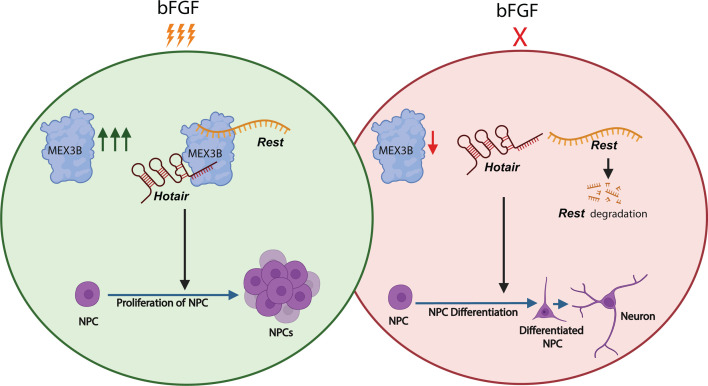

## 1. Introduction

Among the numerous post-translational modifications regulating the cellular proteome, ubiquitination stands out because of its ability to control protein turnover rates by marking proteins for degradation. A tripartite cascade of enzymes is involved in the ubiquitination process: the ubiquitin-activating enzyme E1, the ubiquitin-conjugating enzyme E2 and the E3 ubiquitin ligase. E3 ligases constitute the last step of the ubiquitination process, wherein they catalyse the transfer of ubiquitin to specific substrates. The vast number (approx. 1000) and diversity of E3 ligases [1–4] present in eukaryotes allow them incredible versatility in the regulation of protein fates, including protein stability [4–6], the subcellular distribution of proteins [7] and protein degradation either via the proteasomal machinery [8,9] or by autophagy [10–12]. Such control over the cellular proteome bestows E3 ligases with the ability to influence key processes such as cell cycle regulation [13–15], cell migration [16,17], apoptosis [18] and overall mammalian development [19].

Within neurons, E3 ligases influence the neuronal proteome and they are known to control various aspects of neuronal development, including neurogenesis [20–22], neurite development [23–25], regulation of neuronal morphogenesis [26–29], synapse formation [30–32], synapse pruning [33,34] and both Hebbian [35] and homeostatic [31,36,37] forms of synaptic plasticity, among others. Aberrations in E3 ligase functions have been linked to disorders involved in neuronal development [38–40] as well as neurodegeneration [41–44].

Subsets of E3 ligases have alternate identities. This is attributed to the diverse functions of different protein domains present in addition to the standard RING (Really Interesting New Gene), HECT (Homologous to the E6-AP C-terminus) or RBR (RING-between-RING) domains responsible for their function as ubiquitin ligases [3,45]. The ‘moonlighting’ function of these E3 ligases provides an additional level of control over gene expression within the cellular compartments; this is especially important for E3 ligases that contain RNA-binding domains (RNA-binding ubiquitin ligases (RBULs)) [45–48], such as the TRIM family of E3 ligases [46,48,49] and Roquins [47]. The RNA-binding ability of these E3 ligases provides them with additional control of the cellular proteome by dynamically regulating transcription, post-transcriptional and translation paradigms [21–25]. Especially during neurogenesis, the temporal expression of genes is very tightly regulated, and there is heightened necessity for the participation of RBULs, as they are capable of coupling RNA metabolism and protein turnover [45,49]. This, in combination with reports of them being involved in various stages of neuronal development [21–29], led us to investigate how E3 ligases regulate the transition of neuronal progenitor cells (NPCs) from a state of proliferation to that of differentiation. Specifically, we wanted to understand whether the RNA-binding abilities of development-associated E3 ligases provide them with mechanistic advantages to control the expression of genes relevant to driving the proliferation–differentiation balance at the onset of neuronal development.

In this study, we identify that an RNA-binding E3 ligase, MEX3B, promotes the maintenance of proliferative states in NPCs. It belongs to the Mex3 family of RBULs [50,51] and exhibits differential expression when NPCs undergo transition from proliferative to differentiated states. No studies thus far document the involvement of MEX3B in determining the fate of cortical progenitor cells. We find that MEX3B aids in maintaining a proliferative state via the regulation of *Rest* mRNA stability. This is achieved by the formation of a tripartite complex containing MEX3B, *Rest* mRNA and *Hotair* long non-coding RNA. Disruption of this complex following loss of *Hotair* leads to the reduction of *Rest* abundance. Our study provides a post-transcriptional mechanism of NPC proliferation involving the non-canonical function of RBULs.

## 2. Results

### 2.1. Identification of differentially expressed E3 ligases in neural progenitor cells during the transition from proliferative to differentiated states

**Figure 1.**
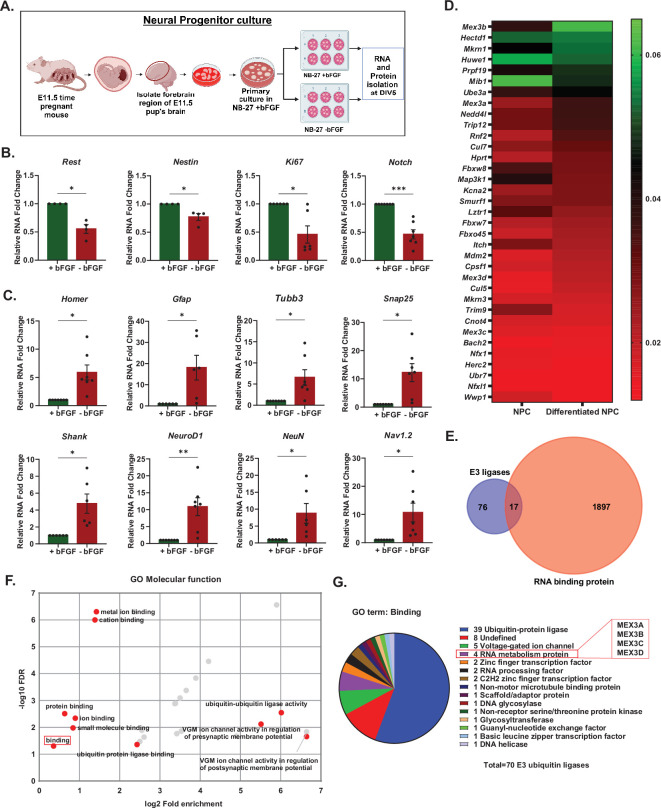
The RNA-binding E3 ubiquitin ligase *Mex3b* is differentially expressed in Neural Progenitor Cell (NPC) cultures. (A) Schematics for obtaining NPC cultures. (B) Differential expression of proliferation markers in differentiating vs proliferating NPCs obtained by qRT-PCR from *n* = 4 to *n* = 7. (C) Expression of differentiation markers in differentiating versus proliferating NPCs obtained by qRT-PCR from *n* = 6 to *n* = 7. All qRT-PCR data are shown as mean ± s.e.m. Statistics were computed using an unpaired *t*‐test with Welch’s correction, *p < 0.05, **p < 0.01, ***p < 0.001. (D) Heatmap representing a subset of the microarray for E3 ligases in proliferating and differentiating NPCs from *n* = 2 (electronic supplementary material, figure S1A). (E) Venn diagram between E3 ligases from microarray and the RNA-binding proteins reported in a previous study. (F) GO analysis for the molecular function of all E3 ligases in the microarray data (electronic supplementary material, figure S1A). (G) Pie chart representation of all the PANTHER protein classes under the GO term ‘Binding’. Also see electronic supplementary material, figure S1 and data table S1.

**Figure 2.**
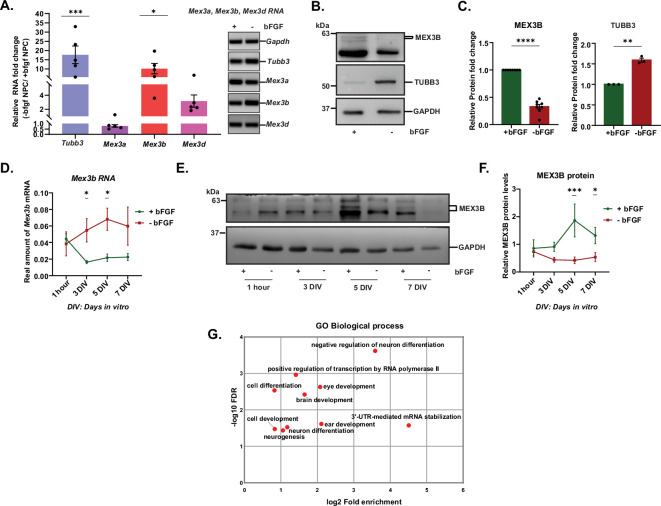
Expression of *Mex3b* mRNA and MEX3B protein during neuronal proliferation. (A) qRT-PCR of the MEX3 family of E3 ligases in NPCs; n = 5. Expression levels of select candidates validated in agarose gel. One-way ANOVA and Fisher’s least significant difference (LSD). (B) Representative western blot of MEX3B (*n* = 8) and TUBB3 (*n* = 3) in differentiating versus proliferating NPCs. (C) Quantification of (B). Unpaired *t*-test with Welch’s correction. (D) qRT-PCR of *Mex3b* RNA at different time points in NPCs in the presence or absence of bFGF; *n* = 6. Two-way ANOVA with Fisher’s LSD. (E) Representative western blot of MEX3B at different time points in NPCs in +bFGF and −bFGF conditions. (F) Quantification of (E) *n* = 6. Statistics were computed by two-way ANOVA with Fisher’s LSD. (G) GO analysis for biological process from MEX3B targets obtained from microarray data from previous studies. *p < 0.05, **p < 0.01, ***p < 0.001, ****p < 0.0001. Also see electronic supplementary material, figure S2 and data table S1.

Neural progenitor cells (NPCs) from E11.5 embryos of timed-pregnant CD1 mice were used to identify E3 ligases that were differentially expressed during the proliferative and differentiation stages of neurons. NPCs derived from CD1 embryos were cultured in neurobasal media in the presence or absence of basic fibroblast growth factor (bFGF) (figure 1A) following established protocols [52,53]. The expression of proliferation and differentiation markers was analysed under bFGF-supplemented and bFGF-deprived conditions at 5 days *in vitro* (DIV5) to evaluate the efficacy of NPC cultures as a model system (figure 1B,C). In the absence of bFGF, there was a significant decrease in the expression of transcripts that indicate proliferation (proliferation markers), such as *Ki67*, *Nestin*, *Rest* and *Notch* compared to the bFGF-supplemented condition (figure 1B; see electronic supplementary material, data table S1). Conversely, there was elevated expression of multiple transcripts supporting differentiation, including *Homer*, *Gfap*, *Tubulin β-III* (*Tubb3*), *Snap25*, *Shank*, *NeuroD1*, *NeuN* and *Nav1.2* (figure 1C; also see electronic supplementary material, data table S1). Our results indicate that the development of NPCs under the influence of bFGF is a valid reductionist model to study the role of E3 ligases in neuron development, specifically during neuronal proliferation and differentiation. Next, we performed microarrays from total RNA extracted from NPCs grown with or without bFGF to identify differentially expressed E3 ligase transcripts, followed by PANTHER Gene Ontology (GO) analysis. Several E3 ligases showed altered expression (figure 1D; electronic supplementary material, figure S1A), with the MEX3 and UBE3 families being the most prominently affected. GO analysis revealed that within the GO molecular function category, the term ‘binding’ was significantly enriched (fold enrichment = 1.28; false discovery rate (FDR) = 0.0498) (figure 1F). Furthermore, detailed classification revealed that MEX3 proteins were associated with both ‘binding’ and the ‘RNA metabolism’ protein class (figure 1G). We cross-referenced known RNA-binding proteins (RBPs) [54] with the E3 ligases identified in the microarray, identifying a compendium of 17 E3 ligases with RNA-binding properties. These were classified as RBULs, with all MEX3 (Muscle Excess 3) family members included (figure 1E; electronic supplementary material, table S1). We selected transcripts of the MEX3 family (*Mex3a*, *Mex3b* and *Mex3d*) for further validation of the microarray data using qPCR. *Tubb3* was used as a differentiation marker (figure 2A). Significant upregulation of *Tubb3* (16.65 ± 4.773-fold, *p* < 0.0001) and *Mex3b* (9.107 ± 0.0129-fold, *p* = 0.0129) was observed under bFGF-deprived conditions. In contrast, *Mex3a* (0.2029 ± 0.1203-fold, *p* = 0.9532) and *Mex3d* (2.167 ± 0.8618 fold, *p* = 0.5326) showed no significant change. Based on the expression profiles of E3 ligases in NPCs and their GO analysis, *Mex3b* emerged as a compelling candidate potentially influencing neuronal differentiation, warranting further investigation.

### 2.2. MEX3B protein and *Mex3b* mRNA have inverse expression patterns during neural proliferation

The expression of MEX3B protein in NPCs was tested in the presence or absence of bFGF. TUBB3 (TUBULIN B-III) was used as a differentiation marker and GAPDH was used for normalization. Surprisingly, MEX3B protein was significantly downregulated (0.6714 ± 0.04210-fold, *p* < 0.0001) in NPCs with no bFGF supplementation (NB-27 −bFGF) compared to those maintained in bFGF-supplemented medium (NB-27 +bFGF) (figure 2B,C; electronic supplementary material, data table S1). The reciprocal expression patterns between *Mex3b* mRNA and its encoded protein MEX3B during neuronal proliferation have been reported in a recent study in *Xenopus* by Takada *et al.* [55]. The study highlights a mechanism of post-transcriptional autoregulation of *Mex3b* mRNA by its encoded protein. MEX3B binds to its own mRNA in a 3′ untranslated region (UTR)-dependent manner, thereby accounting for the opposite expression patterns.

To evaluate whether MEX3B expression in NPCs undergoes similar autoregulatory dynamics in our system, we tested the temporal expression levels of *Mex3b* mRNA and MEX3B protein in NPCs at 1 h, 3 days, 5 days and 7 days in bFGF-supplemented and bFGF-depleted conditions (figure 2D–F). The trajectories of expression of MEX3B protein and *Mex3b* mRNA were analysed both within each experimental condition (i.e. +bFGF or −bFGF) and between the two conditions at corresponding time points. In the absence of bFGF, *Mex3b* mRNA levels showed a progressive upregulation from 1 h to DIV3, peaking at DIV5. Conversely, under bFGF-supplemented conditions, *Mex3b* transcript levels significantly decreased from 1 h to DIV3 and plateaued thereafter (figure 2D, green trace). A comparison of the trajectories of *Mex3b* expression between +bFGF and −bFGF conditions reveals significantly higher levels of the transcript in the latter at DIV3 and 5, respectively (figure 2D; see electronic supplementary material, data table S1). The expression of *Mex3B* mRNA in NPCs was also recapitulated in CD1 mice across key developmental time points (in E11.5 and E15 embryos and in postnatal stages P0, P7, P14 and P28) (electronic supplementary material, figure S2A).

In contrast, the expression of MEX3B protein in +bFGF conditions remained relatively stable from 1 h to DIV3 but showed a marked increase at DIV5. Under bFGF-depleted conditions, MEX3B protein levels remained relatively low but uniform across all time points (figure 2E,F; see electronic supplementary material, data table S1). Comparison of MEX3B protein expression between +bFGF and −bFGF groups revealed a progressive increase in MEX3B from DIV3 to DIV7 in the +bFGF conditions, with the peak expression happening at DIV5 (figure 2E,F; see electronic supplementary material, data table S1). Overall, under +bFGF conditions, MEX3B protein and its mRNA exhibit inversely correlated expression patterns, while no significant difference between the two is observed upon bFGF depletion, supporting the hypothesis that bFGF modulates MEX3B expression post-transcriptionally in NPCs. Our findings are consistent with prior reports [55]. Since proteins are considered as the final functional entities in any cellular context, we decided to focus on MEX3B protein in neuronal proliferation. As the expression of MEX3B was maximum at DIV5 in +bFGF condition, and the difference in MEX3B protein and *Mex3b* mRNA was also the highest, we have selected the same time point for further experiments.

To gain further insights into the plausible physiological roles of MEX3B, we did a gene ontology (GO) analysis on MEX3B targets obtained from a previous study [55]. PANTHER GO analysis of MEX3B targets shows the enrichment of several GO biological processes like ‘negative regulation of neuronal differentiation’, ‘brain development’, ‘neurogenesis’, ‘3′ UTR-mediated mRNA stabilization’, etc. (figure 2G; electronic supplementary material, figure S2B,C).

### 2.3. MEX3B loss of function disrupts the bFGF-driven expression of proliferation markers in NPCs

**Figure 3.**
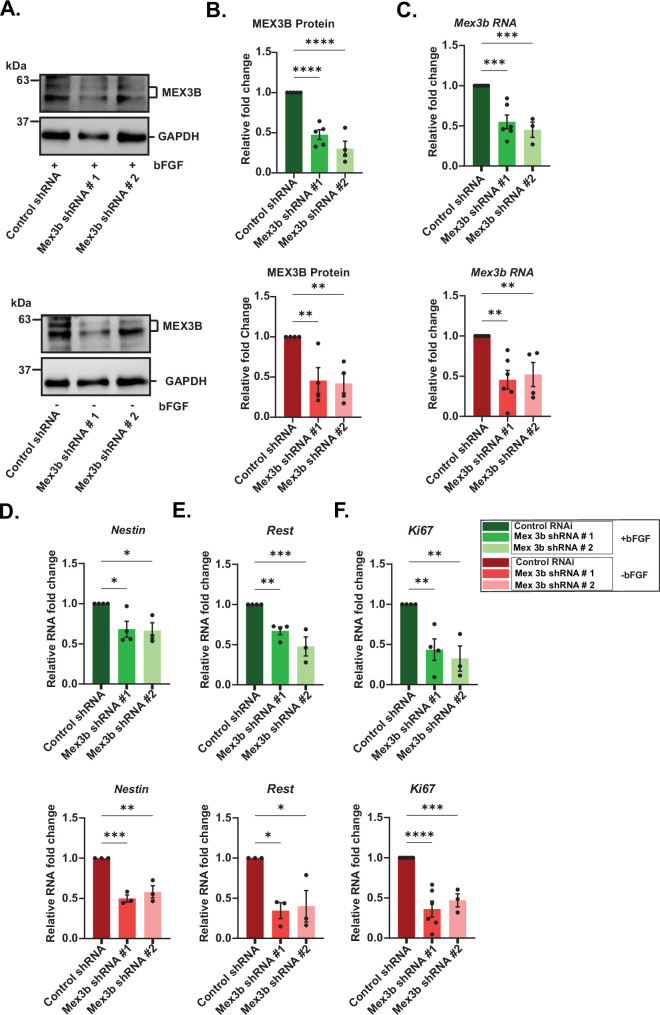
MEX3B RNAi leads to the reduction in proliferation markers in the presence of bFGF. (A) Western blot of MEX3B in NPCs grown in bFGF-depleted or supplemented media. Two shRNA constructs were used for MEX3B RNAi, as indicated. (B) Quantification of (A) *n* = 3–5, **p < 0.01, ****p < 0.0001. (C) qRT-PCR-based quantification of *Mex3b* RNA in NPCs expressing shRNA as indicated, in the presence and absence of bFGF. *n* = 3–6. (D–F) Differential expression of proliferation markers in MEX3B RNAi NPCs in bFGF-supplemented or bFGF-depleted medium: (D) *Nestin*, (E) *REST* and (F) *Ki67*. *n* = 3–6, *p < 0.05, **p < 0.01, ***p < 0.001, ****p < 0.0001. All data are shown as mean ± s.e.m. One-way ANOVA with Fisher’s LSD was used to compute statistical significance. Also see electronic supplementary material, figure S3 and data table S2.

To investigate whether MEX3B regulates proliferation or differentiation in NPCs, we cloned two independent shRNA constructs targeting *Mex3b* transcript into a lentiviral vector co-expressing GFP. Following transduction into NPCs, successful MEX3B RNAi was evaluated for *Mex3b* RNA by qRT-PCR and MEX3B protein by western blot at DIV5 (figure 3A–C; see electronic supplementary material, data table S2).

In addition, the transduced NPCs were analysed at DIV5 to assess changes in key transcripts associated with proliferation and differentiation in bFGF-supplemented or bFGF-depleted conditions. There was a distinct downregulation of proliferation-associated markers in NPCs with MEX3B RNAi, *Ki-67*, *Rest* and *Nestin* transcripts were significantly reduced as compared to Control RNAi in both conditions (figure 3D–F; see electronic supplementary material, data table S2). Transcript levels of differentiation markers remained unchanged (electronic supplementary material, figure S3B,C). Our data suggest that in NPCs, MEX3B is likely to promote the expression of mRNAs encoding proliferation markers.

### 2.4. MEX3B knockdown is sufficient to reduce the pro-proliferative state of NPCs

**Figure 4.**
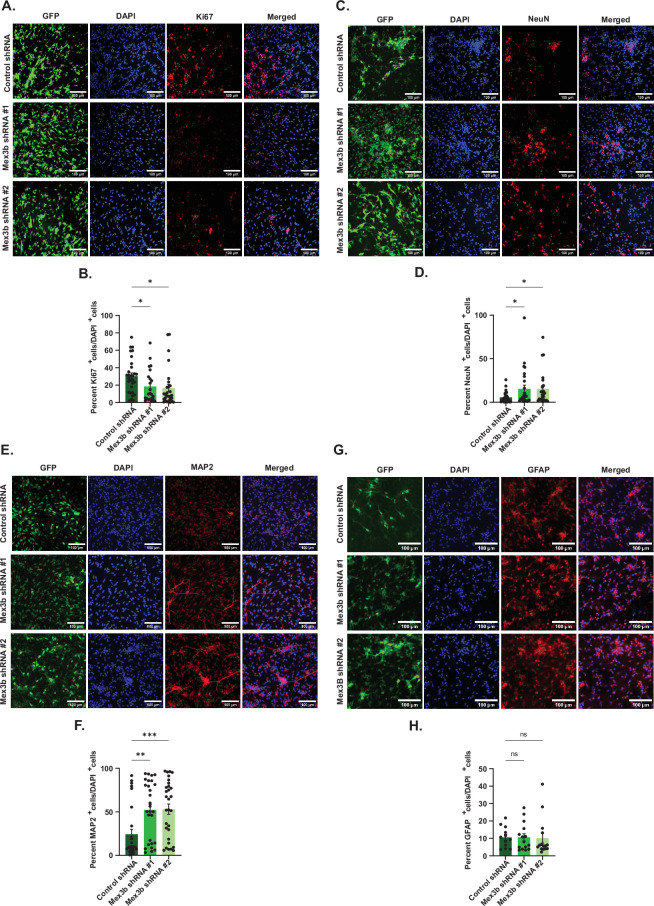
MEX3B RNAi regulates the distribution of proliferation versus differentiation-primed NPCs. (A, C, E, G) Representative photomicrographs of NPCs expressing the indicated shRNA, immunostained with antibodies against (A) Ki67, (C) NeuN, (E) MAP2 and (G) GFAP. Quantification of Ki67**^+^**, NeuN**^+^** and MAP2**^+^** cells are in (B), (D) and (F), respectively, represented as a percentage of total DAPI**^+^** cells. *n* = 23–30 from three biological replicates. (H) Quantification of GFAP**^+^** cells as a percentage of DAPI cells represented in (G). *n* = 12–15, ns = not significant. All data are shown as mean ± s.e.m. One-way ANOVA with Fisher’s LSD is used for computing statistical significance. Scale bar = 100 µm. Also see electronic supplementary material, data table S2.

We wanted to visualize whether the decrease in the transcript levels of pro-proliferative markers upon MEX3B RNAi is also reflected in the distribution of proliferation-primed and differentiation-primed cells within the NPC population, and if this distribution was influenced by bFGF. Following the loss of function of MEX3B, NPCs were immunostained with antibodies against prominent proliferation and differentiation markers. The number of Ki67**^+^**, NeuN**^+^**, MAP2**^+^** and GFAP**^+^** cells in MEX3B RNAi NPCs in both +bFGF and −bFGF conditions were quantified and represented as a percentage of the total DAPI**^+^** population (electronic supplementary material, figure S4A). There was a significant reduction in the percentage of Ki67**^+^** cells, while the percentage of NeuN**^+^**cells and MAP2**^+^**cells increased significantly upon MEX3B RNAi even in the presence of bFGF (figure 4A–F; see electronic supplementary material, data table S2). Ki67 is a marker of proliferation, while NeuN and MAP2 represent neuronal differentiation. Furthermore, no change was observed in the levels of the glial marker GFAP (figure 4G,H; see electronic supplementary material, data table S2). Collectively, observations from figures 3 and 4 suggest that MEX3B knockdown causes a reduction of proliferative-primed cells despite the presence of bFGF, which is known to provide a strong proliferative drive to NPCs. Loss of MEX3B drives neural progenitors to attain neural fate without affecting their glial potential, probably by upregulating neuron-specific differentiation programs.

### 2.5. Loss of MEX3B promotes neurite growth in differentiated neurons even in the presence of bFGF

**Figure 5.**
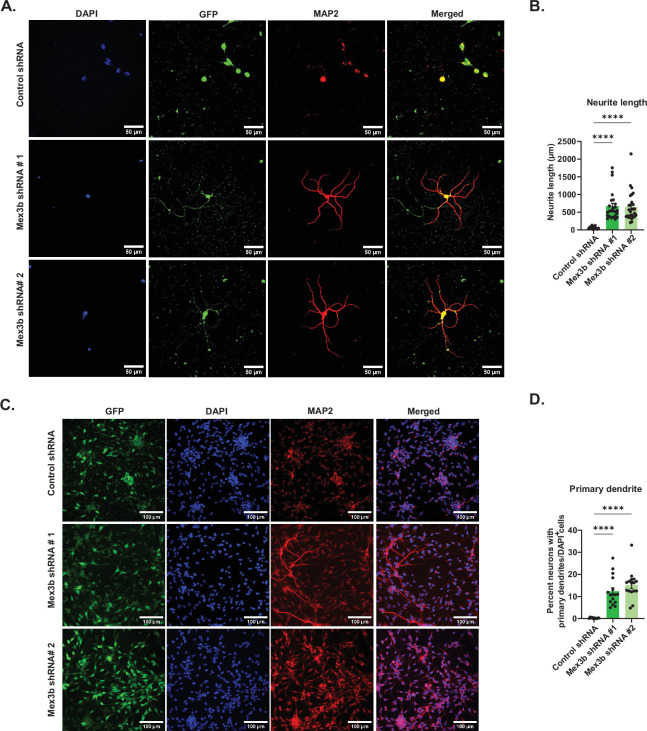
MEX3B knockdown promotes differentiation in bFGF-supplemented NPCs. (A,C) Representative images of MEX3B RNAi or control RNAi NPCs expressing GFP. These NPCs were immunostained with MAP2 antibody and co-stained with DAPI. Scale bar as indicated. (B) Quantification of (A); *n* = 14–25. (D) Quantification of (C); *n* = 11–16, ****p < 0.0001. All data are shown as mean ± s.e.m. One-way ANOVA with Fisher’s LSD is used for computing statistics. Also see electronic supplementary material, figure S4 and data table S2**.**

We assessed whether MEX3B induces distinct morphological changes in differentiation-primed NPCs. Undifferentiated NPCs possess a round or elliptical morphology with minimal or no neurites, whereas with the progression of neuronal differentiation, they start to develop primary and secondary neurites along with the expression of differentiation markers like MAP2. Leveraging this distinction, we quantified the proportion of MAP2**^+^** cells bearing primary neurites as a percentage of DAPI**^+^** cells in NPC populations lacking MEX3B, in the presence of bFGF. Indeed, NPCs with MEX3B RNAi showed a significant increase in the number of cells with primary neurites as compared to control RNAi (figure 5C,D; see electronic supplementary material, data table S2). Furthermore, neurite length was found to be significantly enhanced in MEX3B RNAi compared to the control (figure 5A,B; see electronic supplementary material, data table S2). Sholl analysis also revealed increased dendritic complexity within a 10–120 μm radius from the soma following MEX3B knockdown (electronic supplementary material, figure S4B,C). These results indicate that MEX3B negatively regulates neurite outgrowth and morphological maturation in differentiating NPCs, corroborating our previous observations that the primary role of MEX3B is to support proliferation in NPCs.

### 2.6. The MEX3B*–Hotair–Rest* tripartite complex stabilizes *Rest* mRNA in proliferative NPCs

Our observations established that MEX3B is necessary for the maintenance of the proliferative state of NPCs. We therefore reviewed existing literature for mechanistic insights into the regulatory role of MEX3B. Notably, two molecules of significance emerged: REST, a transcription factor that prevents premature differentiation in NPCs and regulates neuronal specification in the developing cortex [52,53,56], and the long non-coding RNA *HOTAIR* (human). The association of *HOTAIR* (human) with MEX3B promotes ubiquitination-dependent proteasomal degradation in human-derived secondary cell lines [57]. *HOTAIR* is also known to be involved in developmental defects [58] and brain disorders [59–61]. Previous studies indicate that REST interacts with the *Mex3b* mRNA [62], whereas *HOTAIR* associates with REST within two regulatory complexes: the Polycomb Repressive Complex 2 (PRC2) and the LSD1/CoREST/REST complex [63]. As per earlier evidence [62] and our data, a possibility emerges that the regulatory role of MEX3B in murine NPCs is exerted through a multimolecular complex consisting of *Hotair* lncRNA (mouse) and Rest (either as protein or as an mRNA). This association between MEX3B and Rest (in either form) therefore warrants further investigation.

**Figure 6.**
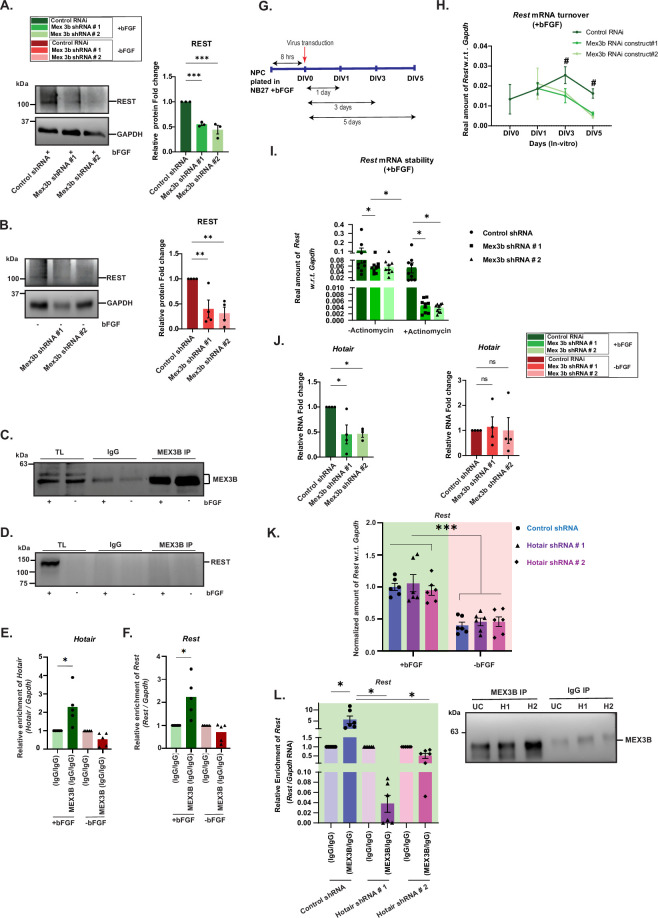
MEX3B protein associates with the lncRNA *Hotair* to stabilize *Rest* in proliferating NPCs. (A,B) Western blot of REST protein in MEX3B RNAi NPCs in +bFGF and −bFGF conditions as indicated. *n* = 3–4. One-way ANOVA with Fisher’s LSD. (C) Western blot of immunoprecipitated MEX3B with or without bFGF. (D) Immunoblot of REST from MEX3B-immunoprecipitated samples. (E,F) Histogram representing the association of *Hotair* lncRNA (E) and *Rest* mRNA (F) with MEX3B. *n* = 5. Unpaired *t*‐test with Welch’s correction. (G) Schematic of *Rest* mRNA stability assay. (H) *Rest* mRNA turnover in NPCs with MEX3B RNAi in the presence of bFGF. (I) *Rest* mRNA stability in NPCs lacking MEX3B in the presence of Actinomycin D, and bFGF. *n* = 7-9. (J) *Hotair* lncRNA abundance in NPCs lacking MEX3B, in +/−bFGF conditions. n = 4. One-way ANOVA with Fisher’s LSD. (K) *Rest* mRNA abundance in NPCs lacking *Hotair* lncRNA, in +/−bFGF conditions. *n* = 5–6. (L) *Rest* mRNA association with MEX3B in NPCs lacking *Hotair* lncRNA in the presence of bFGF. Inset: Immunoblot showing MEX3B immunoprecipitation. Unpaired *t*‐test with Welch’s correction. All data shown as mean ± s.e.m. Two-way ANOVA with Fisher’s LSD is used for computing statistics unless otherwise specified. ^#^*p* = 0.05, *p < 0.05, **p < 0.01, ***p < 0.001. IgG, immunoglobulin G; IP, immunoprecipitation; TL, total lysate. Also see electronic supplementary material, figure S5 and data table S3.

We had two pertinent observations from previous experiments (figures 1B and 3E): first, *Rest* mRNA depletes in the absence of bFGF (figure 1B); and second, MEX3B knockdown in NPCs causes *Rest* mRNA levels to diminish even in the presence of bFGF (that provides a pro-proliferative drive) (figure 3E). To delineate the nature of the association between Rest and MEX3B, we employed a two-step approach. First, we examined how MEX3B RNAi affects the expression of REST protein. As expected, REST protein levels reduced both in the presence and absence of bFGF (figure 6A,B; see electronic supplementary material, data table S3). Second, we immunoprecipitated MEX3B from NPCs grown in bFGF-supplemented or bFGF-depleted media (figure 6C) and assessed the association of REST protein, *Rest* mRNA and *Hotair* lncRNA. Both *Rest* mRNA and the lncRNA *Hotair* (see electronic supplementary material, data table S3) were found to have enhanced association with MEX3B protein in the presence of bFGF, but surprisingly, REST protein was not associated at all (figure 6D–F). Therefore, we focused our attention on MEX3B’s association with *Rest* mRNA rather than REST protein. The turnover of *Rest* mRNA was measured in NPCs with MEX3B RNAi at 1, 3 and 5 days in the presence of bFGF (figure 6G). Depletion of MEX3B resulted in reduced *Rest* mRNA levels as compared to control, suggesting that MEX3B may be involved in either the transcription or the stability of *Rest* (figure 6H). We carried out the same experiments in the presence of Actinomycin D (Act D), a transcriptional inhibitor (figure 6I). Despite blocking transcription, the pattern of *Rest* mRNA turnover in the absence of MEX3B continued unabated, suggesting that MEX3B has a more prominent role in determining the stability of *Rest* transcripts (see electronic supplementary material, data table S3).

Further on, we investigated the effect of MEX3B on the lncRNA *Hotair* in NPCs (figures 6J; electronic supplementary material, figure S5). We found *Hotair* levels to decrease in MEX3B RNAi conditions in the presence of bFGF (see electronic supplementary material, data table S3). However, in the absence of bFGF, *Hotair* levels remained unchanged, suggesting that in the context of bFGF-dependent proliferation of NPCs, *Hotair* is regulated by MEX3B (figure 6J). To identify whether *Hotair* influences *Rest* mRNA, we assessed its expression after *Hotair* RNAi, in the presence and absence of bFGF. Two different shRNA constructs were used against *Hotair* to eliminate off-target effects. *Rest* mRNA levels remained high in the presence of bFGF and decreased in its absence (figure 6K; see electronic supplementary material, data table S3). Indeed, the expression of *Rest* was not influenced by the absence of *Hotair*; rather, its expression pattern recapitulated figure 1B.

How is *Hotair* regulating the association of MEX3B and *Rest*? To evaluate this, we immunoprecipitated MEX3B in NPCs with *Hotair* RNAi in the +bFGF condition (figure 6L). While in control RNAi NPCs, enhanced interaction between MEX3B and *Rest* mRNA was recapitulated as previous; the interaction was abolished in the absence of *Hotair* (figure 6L; see electronic supplementary material, data table S3). The result was consistent for both shRNA constructs of *Hotair.* This observation proves that *Hotair* functions as a scaffold in the MEX3B*–Hotair–Rest* ternary complex; loss of *Hotair* disrupts the complex, and *Rest* dissociates from MEX3B.

Holistically, the observations from this study lead us to postulate that the MEX3B*–Hotair–Rest* tripartite complex is integral to the maintenance of the proliferative capacity in progenitors.

## 3. Discussion

This study highlighted the role of the E3 ubiquitin ligase MEX3 family member, MEX3B, as an RBP in the proliferation of neural progenitors. While E3 ligases have been very well characterized in various contexts of neuronal proliferation and development [20–25], what remains to be comprehensively studied is how a subset of E3 ligases, possessing RNA-binding domains (RBULs), influence the post-transcriptional states of mRNAs involved in the determination of cellular fate. RBULs become interesting candidates to study because the dual possession of ubiquitination and RNA-binding properties allows them to have control over RNA metabolism as well as protein turnover [64]. The function of RBULs, therefore, becomes especially relevant in contexts of neuronal proliferation and differentiation where there exists a large-scale dynamicity in the temporal patterns of gene expression. The focus of this study was to identify whether RNA-binding properties of E3 ligases provide any non-canonical advantages to influence proliferation or differentiation events associated with neuronal development. We performed microarrays from mouse cortical NPC cultures grown in the presence or absence of bFGF, a pro-proliferative cue. Cortical progenitor cells maintain their proliferative state in the presence of bFGF; its withdrawal results in the onset of differentiation, marked by the formation of neurites. NPCs, therefore, provide a viable model to investigate the influence of RBULs in proliferation and differentiation. Microarray studies from NPCs grown in the presence or absence of bFGF revealed a subset of differentially expressed E3 ligases. Cross-comparison of the list of RBPs in mice [54] with the total number of E3 ligases in our microarray (76) revealed that 17 of the E3 ligases were RBULs. Further GO analysis (using PANTHER) of the differentially expressed E3 ligases revealed that among them, the ones that were common to the PANTHER class ‘RNA metabolism’ and ‘binding’ were the MEX3 family (figure 1). Single qRT-PCR of the MEX3 family candidates confirmed that *Mex3b* mRNA displays maximum change between proliferative neuronal progenitors and their differentiated forms (figure 2). MEX3B protein’s function, as an E3 ligase through its RING finger domain [51], is well known, but its potential as an RBP remains elusive. MEX-3, a homolog in *C. elegans*, is known to be involved in the germline totipotency [65] and very recently the role of MEX3B in the spatial organization of Sertoli cells to maintain spermatogenesis in the testis in mice has been identified [66]. A homolog of MEX3A has been reported as the regulator of nervous system development in the vertebrate *Xenopus laevis* and the teleost fish *N. furzeri* [67,68]. However, little to none is known about the expression of the *Mex3b* transcript in the context of neuronal proliferation in vertebrates. From the mouse ENCODE transcriptome data, *Mex3b* transcript levels were found to be specifically higher in the whole brain at E14.5 and in the CNS at E11.5, E14 and E18 [69]. Differential expression of *Mex3b* mRNA in microarray data further strengthened our hypothesis that its translated form (MEX3B) is involved with the proliferation/differentiation of neural progenitors (figure 1 and electronic supplementary material, figure S1). Since MEX3B is an E3 ligase and an RBP, we needed to assess whether it would be functionally active during neuronal proliferation. The expression of MEX3B protein was surprisingly found to be significantly high in the +bFGF compared to the −bFGF condition, which is completely opposite to *Mex3b* mRNA levels. Previous studies helped us understand this ambiguity. A study from Masanari Taira’s group shows the autoregulation of *Mex3b* mRNA by its protein in *Xenopus laevis* [55]. MEX3B binds to the 3′ UTR of its own mRNA to destabilize it by regulating the post-transcriptional expression; hence, the protein and RNA have opposite expression patterns at distinct temporal periods during development. Although not E3 ligases, several other proteins regulate their own mRNA, e.g. both ELAV and Mel-N1 in mice increase the stability of their mRNA by competing with destabilizing factors for binding to their own 3′ UTR region [70–72]. Another protein, TTP, shares considerable similarity in the regulation of its own mRNA with MEX3B through binding to AUUUA sequences [73]. We enquired whether such autoregulation of the *Mex3b* mRNA by its protein is valid in vertebrates as well. Accordingly, we assessed MEX3B protein and its mRNA levels across different time points. Interestingly, in the presence of bFGF, *Mex3b* mRNA displays a steep decline in expression levels with time, with a concomitant increase in MEX3B levels (figure 2). This is similar to the autoregulatory effect of MEX3B on its mRNA as reported in *Xenopus* [55]. Further, the high abundance of the *Mex3b* transcript in the embryonic brain *in vivo* (electronic supplementary material, figure S2) recapitulates its expression level in proliferative NPCs (figure 2), suggesting physiological similarities in both the systems. The steep decline of *Mex3b* mRNA levels in mouse cortex at ages P0–P14 is an interesting observation to explore further. Certain parts of the cortex develop during this time, including important developmental events such as eye and ear opening [74]. Our observation bolsters the idea that increased expression levels of MEX3B protein facilitate proliferation over differentiation in NPCs. Hence, we focused on characterizing the role of the MEX3B protein in the maintenance of the proliferative state of neural progenitors.

Expression of transcripts indicating proliferation, like *Ki67*, *Nestin*, and *Rest,* reduced significantly upon MEX3B knockdown even in the presence of bFGF, indicating that the absence of MEX3B pushes the fate of neural progenitors towards differentiating cells, circumventing the influence of bFGF (figure 3; electronic supplementary material, figure S3). Ki67 is a generic proliferative marker and not neuron-specific, but Rest and Nestin are specific to neural progenitor proliferation. REST protein is considered to be a key transcription factor that enables fate determination of neural progenitors [53,56]; it inhibits the transcription of differentiation markers to maintain the proliferative state of the neural progenitors [52,56]. No change in the transcriptional expression of differentiation markers was observed (electronic supplementary material, figure S3). Therefore, it stands to reason that unlike REST, MEX3B does not influence the transcriptional expression of differentiation markers. Although MEX3B may act as a pro-proliferative cue, the proliferative drive of bFGF is stronger. Consequently, MEX3B RNAi alone is not sufficient to promote the transcription of differentiation factors by circumventing bFGF.

Immunocytochemistry data reveal that upon MEX3B knockdown, the number of cells expressing Ki67 protein goes down while the number of cells expressing MAP2 and NeuN increases. MAP2 and NeuN are expressed in fully differentiated, mature neurons (figure 4) [75,76]. In the presence of bFGF, MEX3B RNAi resulted in a significant increase in the percentage of cells with primary neurites, as well as neurite length. Together, these are indicators of a greater proportion of differentiated cells present in the pool of progenitors. Along with neurite length, changes were also observed in dendritic arborization following the loss of function of MEX3B (figure 5; electronic supplementary material, figure S4). The collective inference from our data is that MEX3B represses neuronal differentiation in a bFGF-dependent manner.

**Figure 7.**
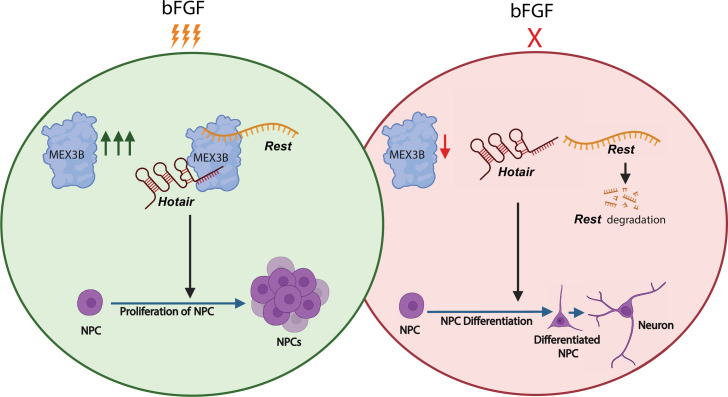
Representative model of bFGF’s influence on the formation of the MEX3B*–Hotair–Rest* tripartite complex. In the presence of bFGF (green circle), the tripartite complex comprising MEX3B, *Rest* mRNA and *Hotair* promotes NPC proliferation. Within NPCs deprived of bFGF (red circle), MEX3B dissociates from *Hotair* and *Rest,* which may result in the degradation of *Rest* mRNA and the concomitant transition from proliferation to differentiation stages.

Now, several hypotheses may be posited on how MEX3B influences neural progenitor fate. We saw a significant change in both *Rest* mRNA and REST protein upon MEX3B knockdown (figure 6; electronic supplementary material, figure S5), prompting further investigation of the RNA-binding behaviour of MEX3B. The human orthologue of *Hotair*, namely *HOTAIR,* has previously been reported to interact with MEX3B and other E3 ligases to facilitate the ubiquitin-mediated degradation of its target protein Snurportin [57]. MEX3B has also been reported to ubiquitinate other targets like RUNX3 in association with *HOTAIR* [77]. *HOTAIR* interacts with REST, resulting in transcriptional inactivation or gene silencing [63]. Furthermore, a database released by the Ma'ayan Laboratory of Computational Systems Biology shows target genes of the REST transcription factor in low- or high-throughput functional studies from the CHEA Transcription Factor Targets dataset. *Mex3b* mRNA is one of the targets mentioned among 4675 others [62]. Based on our observations and previous literature, we speculated that in NPCs, MEX3B may interact with *Hotair* and REST/*Rest*. To test our model, we immunoprecipitated MEX3B and evaluated the association of *Hotair* and *Rest* transcripts in NPCs along with assessing MEX3B-REST protein interaction. Further, we also analysed the transcript levels of *Rest* following the knockdown of *Hotair* lncRNA or MEX3B protein in the presence or absence of bFGF. Results from our immunoprecipitation experiments confirmed a direct interaction between MEX3B protein, *Hotair* and *Rest mRNA* in proliferative NPCs (maintained in bFGF). MEX3B had no interaction with REST protein (figure 6). Our data corroborate that MEX3B exerts control specifically on *Rest* mRNA. The loss of MEX3B in proliferative NPCs led to a reduction in *Rest* mRNA levels, which may account for the reduced REST protein levels observed in MEX3B RNAi (figure 6). Further, our experiments involving the transcriptional inhibitor Actinomycin D revealed a pronounced reduction of *Rest* under transcriptional blockade, supporting the conclusion that MEX3B directly contributes to the stabilization of *Rest* mRNA in proliferative NPCs (figure 6). Altogether, these observations strengthen our proposed model (figure 7) that MEX3B forms a tripartite complex with *Hotair* lncRNA and *Rest*, thus stabilizing *Rest* mRNA.

The knockdown of *Hotair* has no effect on *Rest* abundance in proliferative NPCs (figure 6). Notably, we found MEX3B to be a regulator of *Hotair*, because the loss of MEX3B reduced *Hotair* levels. Unlike MEX3B, the loss of *Hotair* function had no effect on *Rest* mRNA levels. Our data demonstrate that *Hotair* is necessary but not sufficient for MEX3B-dependent *Rest* stability in proliferative NPCs. Immunoprecipitation of MEX3B in the *Hotair* RNAi background within NPCs resulted in the abolishment of the MEX3B*–Rest* association (figure 6L), suggesting that *Hotair* acts as a facilitator of the interaction between MEX3B and *Rest.* Since there was no observed reciprocity in the regulation of *Hotair* and *Rest* mRNA, but both of them had differential expression upon MEX3B RNAi in bFGF-supplemented NPCs, we arrived at three conclusions: first, MEX3B is necessary and sufficient for the regulation of *Rest* mRNA in the presence of bFGF; second, *Hotair* does not have an overt regulatory effect on MEX3B protein and *Rest* mRNA but is physically interacting with both; and third, bFGF is critical to establish this tripartite association. The identity of other proteins that participate in the MEX3B*–Hotair–Rest* complex is unknown at this point.

Our study opens a new avenue for the post-transcriptional regulation of neural fate determination through the regulatory control of REST by an RBUL MEX3B. It also provides a link between an lncRNA, an RBUL protein, and the transcription factor Rest in proliferative neural progenitors. We explored the non-canonical functions of MEX3B and tried to elucidate how the complex interplay between RNAs and RBPs may dictate cellular phenotypes. This study paves the way towards identifying how RBULs play divergent roles in regulating neuronal development and aids us in the treatment of neurodevelopmental disorders caused by defects in neuronal proliferation and differentiation.

## 4. Conclusion

MEX3B belongs to a special class of E3 ubiquitin ligases that also possess RNA-binding motifs. Our study provides an in-depth characterization of MEX3B’s influence on the proliferative properties of NPCs. Structural changes in proliferating NPCs, attainment of neuronal fate and the display of characteristics similar to differentiated neurons upon MEX3B RNAi further underline its involvement in neural fate determination. From the observations obtained from our study, we can conclude that MEX3B brings about a strong pro-proliferative drive that aids in the occlusion of differentiation programs in NPCs. MEX3B associates with the master switch Rest (mRNA) along with an lncRNA *Hotair*, known for its role in neuronal development. Overall, the study emphasizes that the MEX3B*–Rest–Hotair* axis is integral to a complex cascade of events that maintain neural progenitors in a proliferating phase. We highlighted that the maintenance of this proliferative state requires *Rest* mRNA stability via the tripartite axis. Our study provides a hitherto uncharacterized example of an E3 ligase using its RNA-binding property to spatiotemporally regulate an intricate molecular network involving post-transcriptional mechanisms underlying neural development.

## 5. Material and methods

### 5.1. Animal maintenance

Time-pregnant female CD1 mice were used for NPC cultures. Animals were obtained from the in-house National Brain Research Center Animal house facility. Animals were maintained at room temperature (25 ± 0.5°C), humidity was 40 ± 5% with a 12 h light/dark cycle with food and water provided *ad libitum,* and mating pairs were set as per requirement. The day of vaginal plug was observed as embryonic day 0 (E0). Pregnant dams were maintained till E11.5.

### 5.2. Neural progenitor cell culture

NPC culture was done from E11.5 embryos following previous protocol [52,53]. Briefly, 100 mm dishes were coated with PDL (50 µg ml^−1^) and Laminin (5 µg ml^−1^) and incubated overnight. The following day the dishes are washed and dried. 7.5 ml of Neurobasal (#21103049) supplemented with B27 (#17504044) and bFGF (40 ng ml^−1^) (NB-27 +bFGF) media was added to the dishes. E11.5 embryos were dissected from a timed-pregnant dam into dissection media; the forebrain from each of the embryos was dissected out and kept in NB-27 +bFGF media. Triturated gently in 1 ml NB-27 +bFGF in a 1.5 ml microcentrifuge tube to obtain 4 to 8 celled clumps. Five hundred microlitres of neurosphere/cell suspension was added to the tube containing 4 ml fresh NB-27 +bFGF and the resultant cell suspension was plated onto the two 100 mm dishes. The culture was maintained until it reached confluency by changing half the media every alternate day. On day 7, the neurospheres were dissociated using 0.25% trypsin; DMEM with 10% fetal bovine serum (FBS) was added, and cells were centrifuged at 800 rpm at room temperature to remove trypsin. Resuspended in fresh NB-27 +bFGF media. The cells were visualized and counted using a haemocytometer and plated at 150 000 cells per well (35 mm diameter) on 6 well plates. Initially, all cells were plated in NB-27 +bFGF media after 8 h, the media for −bFGF wells was changed to NB-27 without bFGF.

### 5.3. Microarray data and gene ontology analysis

NPC cultures with bFGF and without bFGF were grown as described above for microarray analysis. The microarray data obtained were normalized with GAPDH, and the heatmap was made using GraphPad Prism 9.

Gene ontology analysis was performed for all the E3 ligases present in the microarray data and for the Mex3B targets obtained from previous studies (see supplementary table S1 of [55]) using the PANTHER Database (http://pantherdb.org) [78]. Panther statistical over-representation analysis was performed with a statistical test type chosen to be Fisher’s exact and corrected for FDR. The fold enrichment of the transcripts was generated for annotated datasets including GO: Biological Process, GO: Cellular Components and GO: Molecular Function, along with FDR values. Significantly enriched GO terms were analysed and plotted in GraphPad Prism 9. The Venn diagram was made using online available tools [79] to identify proteins common to RNA-binding proteins reported so far (see supplementary table S2 of [54]) and the E3 ubiquitin ligases used in our microarray data.

### 5.4. Lentivirus preparation

Prior to lentivirus preparation, shRNAs were designed against Mex3b, as shown in table 1.

The shRNAs were cloned into the pLVTHM plasmid (Addgene #12247) at MluI and ClaI sites. Lentiviruses were generated by co-transfecting 20 µg of the transfer vector (mCherry cassette under the EF1α promoter and an shRNA cassette against Mex3b or non-targeting control under the H1 promoter in the pLVTHM plasmid), 15 µg of psPAX2 (Addgene plasmid #12260) and 6 µg of pMD2.G (Addgene plasmid #12259) into HEK293T cells. HEK293T cells were cultured in low-glucose DMEM media (Gibco) with 10% FBS (Gibco) at 5% CO_2_ and 37°C. Transfection of 2 × 10^6^ of HEK293T cells was performed using the calcium phosphate method. Following transfection, the media containing the transfection mixture was replaced with fresh media after 8 h. Culture media containing lentivirus particles was collected 72 h post-transfection, and the viruses were concentrated through ultracentrifugation and resuspended in NB-27 media. Viral titres were determined by infecting HEK293T cells and counting the cells using the haemocytometer, with titres ranging from 1 to 3 × 10^7^ TU ml^−1^.

**Table 1.** List of shRNAs designed.

S. no.	targets	shRNA antisense sequence (5′____3')
1	UC shRNA	ATCTCGCTTGGGCGAGAGTAAG
2	Mex3b shRNA #1	ACTTGGTGAAGCTGAGTTCCC
3	Mex3b shRNA #2	AGAAAGCTGACTGAATCCGCC
4	Hotair shRNA #1	GCCCAGATTTAGAGACAATGG
5	Hotair shRNA #2	GGGACTGAGAGGCACTAATTA

RNA interference (RNAi) was carried out by infecting NPCs at DIV0 (days *in vitro*), which is 8 h post plating, with lentivirus expressing shRNAs targeting the Mex3b and the non-targeting control. Fifty per cent of the media was changed every alternate day. Viral infection was tracked by observing GFP expression in infected cells. The efficiency of the knockdown was confirmed by isolating RNA and protein from Mex3b and non-targeting shRNA virus-infected cultures at DIV5 using Trizol and 1× RIPA buffer, respectively, and further processing the samples as mentioned below.

### 5.5. RNA isolation and qRT-PCR analysis

At DIV5 post-NPC culture, samples from 3 to 6 wells of a 6 well plate were pooled to isolate RNA from an equal number of cells for each experimental condition. Cells were washed with 1× phosphate buffer saline (PBS) to remove media completely, and RNA isolation was done using Trizol reagent. Samples were DNase treated using TURBO DNase (Invitrogen #AM2238). cDNA was prepared using an Invitrogen Superscript-III kit (#18080093), and qRT-PCR was performed using SYBR green (Applied Biosystems #4367659). qRT-PCR results were analysed by the δδCt method. See also table 2.

**Table 2.** List of qRT-PCR primers

S. no.	name of gene	species	forward primer	reverse primer
1	Mex3b	mouse	TTTGTGGGTGGGTTCCTACAGC	AACTCCTGGCCCTTCCATGAAC
2	Hotair	mouse	GGCTTCTGACTGCTAAGTGTTAGG	CAGGAAGGGCAAAGGCTTCATTC
3	Rest	mouse	GCGGAAGACAAATGCAGGA	TTCGGCTTCGTACTGGCAA
4	Ki67	mouse	ATCATTGACCGCTCCTTTAGGT	GCTCGCCTTGATGGTTCCT
5	Nestin	mouse	CCACATGATTAAGAGCCTGCTAG	TAGATGGTTCACAATCCTCTGGT
6	Notch1	mouse	CCCTTGCTCTGCCTAACGC	GGAGTCCTGGCATCGTTGG
7	Homer	mouse	GGATTCTCCTCTGAGCATCA	ACTGGTCAGCTCCATCTTCT
8	Gfap	mouse	ATCTATGAGGAGGAAGTTCGAG	CTGTCTCTTGCATGTTACTGGT
9	Tubulin β-III	mouse	GCGCCTTTGGACACCTATTC	CCGCGCCCTCCGTATAGTGC
10	SNAP25	mouse	GAGCTGGAGGAGATGCAGAG	CTCTTCAACCAGCTGTAGCAT
11	Shank	mouse	TCGAGGACCATGAGATCGAA	CGCACGCTCGATGTTCAT
12	NeuroD1	mouse	CAAGGTGGTACCTTGCTACTC	CGCAGGATCTCTGACAGAGC
13	NeuN	mouse	AACCACGAACTCCACCCTTC	GACCTCAATTTTCCGTCCCTC
14	Nav1.2	mouse	GGCACAATCAGTGCTGGTACC	CAGCAAGGGATTCCCTGGT
15	Gapdh	mouse	CGTATTGGGCGCCTGGTCAC	CGGCCTCACCCCATTTGATG

For the physiological relevance of Mex3b, CD1 mice at different developmental time points, viz. (embryonic days) E11.5, E15 (postnatal days) P0, P7, P14, P21, P28, were sacrificed. The cortex was isolated from them and homogenized in Trizol, which was used to isolate RNA. cDNA was prepared, and qRT-PCR was performed as above.

For time-point experiments samples from 3 to 6 wells of NPCs grown in 6 well plates were collected at 1 h and on days 3, 5 and 7 days from the time point of changing the media from NB-27 with bFGF to NB-27 without bFGF. Cells were washed with 1× PBS to remove media completely and collected in Trizol reagent. RNA and protein both were isolated from the same Trizol-collected cells. RNA and protein were analysed by qRT-PCR and western blot, respectively.

All qRT-PCR primers have been listed in table 2.

### 5.6. mRNA stability and mRNA turnover assay

The stability of *Rest* mRNA and its turnover was using actinomycin D (transcription blocker). Viral transduction was done 8 h after plating and incubated for 3−5 days. Actinomycin D dissolved in DMSO (10 µg ml^−1^) was added to the NPCs and incubated at 37°C, 5% CO_2_ for 3 h. Cells were collected in Trizol post 3 h after giving one wash with 1× PBS. For conditions without actinomycin D, an equivalent amount of vehicle was added to the cells. Collected cells were subjected to RNA isolation by Trizol and qRT-PCR was done as before. The time-points selected for the mRNA turnover experiment are shown in figure 6G.

### 5.7. Protein isolation and western blot

NPC cultures were lysed using 1× RIPA buffer for western blot analysis and protein estimation performed by bicinchoninic acid (BCA) assay, and an equal amount of protein was loaded onto 8–10% SDS-PAGE gels. The resolved proteins were then transferred onto nitrocellulose membranes (Millipore), followed by blocking with 5% bovine serum albumin (BSA) in 1× TBST (Merck). The blots were probed with specific antibodies: MEX3B (1 : 500; ThermoFisher, #A301-194A), TUBB3 (TUJ1) (1 : 10 000; Sigma, #T8578), GAPDH (1 : 10 000; Sigma, #G9545) and REST (1 : 500; Millipore, #09-019). GAPDH protein was used for normalization. The protein ladders used in western blot are the BioRad Precision plus protein ladder (#1610374) and the BlueRay Prestained Protein Ladder (#SP006-0500)

### 5.8. Immunocytochemistry

For Immunocytochemistry 20 000 neurospheres were plated in the 12 well plates with 18 mm coverslips coated with poly-d-lysine and laminin. Eight hours post plating, cells were infected with the virus for control RNAi and two constructs of MEX3B RNAi. At DIV6, the coverslips were fixed with fixative buffer (a solution of 2% paraformaldehyde (PFA) and 4% sucrose in 1× PBS) and subjected to immunostaining, following a previously described protocol [80] with minor adjustments.

NPCs were washed three times with PBS and then fixed with 2% PFA and 4% sucrose in 1× PBS at 37°C for 30 min. Coverslips were permeabilized with 0.5% Triton-X-100 in 1× PBS (PBST) for 15 min, and blocking was performed at room temperature in a blocking solution consisting of 5% BSA and 0.1% Triton-X-100 in 1× PBS. The NPCs were subsequently incubated with specific antibodies against MAP2 (1 : 300, mouse, clone K28/43, Neuromab, #75-028), NeuN (1 : 500, goat, Invitrogen, #PA5-143586), Ki67 (1 : 50, mouse, Abcam, #ab279653) and GFAP (1 : 1000, mouse, Millipore, #MAB360) overnight at 4°C. The following day, NPCs were washed three times with 1× PBST (0.01% Triton-X-100) and incubated with the respective secondary antibodies conjugated with Mouse-Alexa-546 (Invitrogen Molecular Probes, #A-11003) or Mouse-Alexa-633 (Invitrogen Molecular Probes, #A-21052) or Rabbit-Alexa-633 (Invitrogen Molecular Probes, #A-21071) or Goat-Alexa-633 (Invitrogen Molecular Probes, #A-21082) at room temperature for 2 h. Following three washes with 1× PBST (0.01% Triton-X-100), coverslips were mounted using Vectashield mounting medium containing DAPI (Vector Laboratory, #H1200) and sealed with clear nail varnish.

### 5.9. Confocal microscopy

Using a Nikon A1HD point scan confocal microscope equipped with a Plan Apo 10× objective with (numerical aperture) NA 0.25, we captured a large image of dimension 11 × 11 frames, 16-bit images of the entire coverslip containing immunostained NPCs with each image at a resolution of 1024 × 1024 pixels, and an optical zoom of 0.7. We acquired 6−12 optical sections with a step size of 3 µm. To facilitate further analysis, the entire coverslip image was divided into multiple non-overlapping equal regions of interest (ROIs).

Fluorescence labelling for DAPI, GFP (lentivirus RNAi), Alexa-546 (GFAP, Ki67) and Alexa-633 (MAP2, NeuN) was achieved by exciting with a 405 nm, 488 nm argon ion laser, and a 561 nm, 640 nm helium-neon (HeNe2) laser, respectively. For representative images, a Plan Apo 40× objective with NA 0.65 was used for imaging by a Nikon AXR point scan confocal microscope. For Sholl analysis, images were taken with a Nikon Plan Apochromat 60× objective with NA 0.8 oil immersion with an optical zoom of 0.7 and at a resolution of 1024 × 1024 pixels. We acquired 6−12 optical sections with a step size of 0.75 µm. Consistent laser power, detector gain, amplifier offset and pinhole diameter settings were maintained for all experiments across all conditions.

### 5.10. Cell counting

Cell counts for MAP2 were performed by an experimenter using the ImageJ plugin 'Colocalization Object Counter' [81]. For the neuronal dendritic marker MAP2, we determined the lowest noise tolerance that would not erroneously identify a cell as MAP2 positive and considered it as background across our images. Auto threshold was applied consistently across all experimental conditions. In the DAPI channels, the auto threshold was set to include all cells displaying any positive fluorescence. The number of DAPI-positive cells and number of MAP2-positive cells were determined in the respective channels for specific ROI. The data obtained were represented as the percentage of MAP2-positive cells with respect to DAPI-positive cells.

The ImageJ plugin ‘Analyze Particles’ was used for counting Ki67, NeuN and GFAP-positive cells. Images were processed accordingly by making maximum intensity projections, adjusting the threshold, and converting the image to 8-bit within ImageJ. The best autothreshold type was determined and set for each channel for Ki67, NeuN and GFAP. Corresponding DAPI images were processed similarly. The ImageJ plugin ‘Watershed’ was used to segregate all the cells. A particle size greater than 10 pixels^2^ were considered as one cell. Circularity parameters were set between 0 and 0.1. The number of DAPI-positive cells and the number of Ki67/NeuN/GFAP-positive cells were determined in the respective channels for specific ROI. The data obtained were represented as the percentage of Ki67/NeuN/GFAP-positive cells with respect to DAPI-positive cells. Statistical significance was assessed using a one-way ANOVA test with Fisher’s LSD post hoc test. All analyses were conducted blindly to the experimental conditions.

### 5.11. Counting cells with primary neurites

The ImageJ plugin ‘AutoneuriteJ’ [82] was used to count the number of MAP2-positive cells with primary neurites. MAP2 and DAPI channels were thresholded, and maximum intensity projections were created separately for both channels. Part I of the plugin was used to create the masked images based on the diameter of the nucleus for DAPI staining and the minimal area of neuron provided. Part II of the plugin was used to count the number of neurons based on the specific neurite length parameter and the number of joint neurons. The total number of cells with primary neurites was calculated by adding the number of neurons and the number of joint neurons. The data obtained were represented as the percentage of cells with primary neurites with respect to DAPI-positive cells.

### 5.12. Sholl analysis

We conducted Sholl analysis and neurite length measurements on NPCs that had been infected with lentivirus and immunolabelled with MAP2 antibody, which served as a dendritic marker for assessing neuronal morphology following RNAi. For this analysis, we employed SynD, a software tool developed by Schmitz *et al.* [83]. In this analysis, we first identified the cell soma and proceeded to count intersections within concentric circles, each with increasing radii of 10 µm, originating from the centre of the cell soma. Each image was analysed manually. We then plotted the number of intersections against their respective distances from the centre of the cell soma. Total neurite length for each neuron was also obtained from the same analysis. To assess statistical significance, we compared the results between the non-targeting control RNAi and MEX3B RNAi samples. To do so, we used two-way ANOVA as our chosen statistical method for Sholl analysis.

### 5.13. Immunoprecipitation

MEX3B was immunoprecipitated from NPC cells grown in +bFGF and −bFGF for 5 days following a previous protocol [84] with minor modifications. For *Hotair* knockdown, NPCs were grown in +bFGF. Viral transduction was done after 8 h of plating. Cells were then allowed to grow normally until DIV5 with regular media supplementation. On DIV5 approximately 1.5 million cells (3–6 wells of a 6 well plate) were used for IP, media was removed, and cells were washed with 1× PBS and scraped into Immunoprecipitation buffer (20 mM Tris (pH 7.5), 100 mM KCl, 5 mM MgCl_2_, 0.5% Nonidet P-40, 1 mM DTT, 0.5 µg µ^−1^ Heparin, 100 unit ml^−1^ Superase In RNase Inhibitor, 1 µl ml^−1^ EDTA-free Roche Protease inhibitor cocktail) and homogenized by mild vortexing. The homogenized lysate was centrifuged at 2000 g for 10 min at 4°C, and the supernatant was collected. Protein estimation of lysate was performed using a Bicinchoninic Acid (BCA) assay kit (Thermo Scientific #23227), and equal amounts of protein across all conditions were used for further immunoprecipitation. Protein-G agarose beads were equilibrated with immunoprecipitation buffer (20 mM Tris (pH 7.5), 100 mM KCl, 5 mM MgCl_2_). Lysate was precleared with these equilibrated beads. Precleared lysate was divided into two halves; one half was incubated with MEX3B antibody and the other half with IgG antibody (non-binding control) with continuous mixing at 50 rpm at 4°C for 4 h. Equilibrated Protein-G-agarose beads were added to the antibody-lysate mixture and incubated with continuous mixing at 50 rpm at 4°C for 2 h. After incubation the beads were collected by centrifugation at 8000 *g* for 10 min at 4°C. This matrix of beads was washed with immunoprecipitation buffer followed by high salt buffer (20 mM Tris (pH 7.5), 200 mM KCl, 5 mM MgCl_2_, 0.5% Nonidet P-40, 1 mM DTT, 0.5 µg µl^−1^ heparin, 100 unit ml^−1^ Superase In RNase Inhibitor, 1 µl ml^−1^ EDTA-free Roche Protease inhibitor cocktail) washes and an equilibrating wash. The beads were recovered and subjected to RNA isolation using the Trizol method with the PureLink RNA Mini Kit (Invitrogen), and cDNA was prepared for qPCR. Some part of the beads were kept for protein isolation by boiling in Laemmli buffer to visualize and quantify the result by western blot. Clean blot (Thermo Scientific #21232) was used as a secondary antibody for the western. Antibody used for MEX3B IP (1 : 500; ThermoFisher, #A301-194A) (1 : 100; Santa Cruz, #sc-515833). Western blot for REST was performed from the IP samples using anti-REST antibody (1 : 1000; Invitrogen, MA5−35361).

### 5.14. Statistical analysis

All the data are represented as mean ± s.e.m. Statistical significance was assessed using the unpaired two-tailed *t*‐test with Welch’s correction or by one-way or two-way ANOVA with Fisher’s LSD post hoc test for all qRT-PCR, western blot and imaging data as indicated. A two-way ANOVA test with Fisher’s LSD post hoc analysis along with multiple comparisons was used for the Sholl and mRNA turnover assay as indicated. For GO analysis, Fisher’s exact test was used with FDR value calculations. All the data areas were analysed and plotted using the GraphPad Prism 9. GO analysis statistical values were obtained from PANTHERGO and plotted in the GraphPad Prism 9. The significance threshold was set at *p* < 0.05 for all the experiments.

## Data Availability

Western blot images and raw data for all the plots are accessible from Figshare [85]. Supplementary material is available online [86].

## References

[1] Lescouzères L, Bomont P. 2020 E3 ubiquitin ligases in neurological diseases: focus on gigaxonin and autophagy. Front. Physiol. **11**, 1022. (10.3389/fphys.2020.01022) 33192535 PMC7642974

[2] Yang Q, Zhao J, Chen D, Wang Y. 2021 E3 ubiquitin ligases: styles, structures and functions. Mol. Biomed. **2**, 1–17. (10.1186/s43556-021-00043-2) 35006464 PMC8607428

[3] Jackson S, Xiong Y. 2009 CRL4s: the CUL4-RING E3 ubiquitin ligases. Trends Biochem. Sci. **34**, 562–570. (10.1016/j.tibs.2009.07.002) 19818632 PMC2783741

[4] Iconomou M, Saunders DN. 2016 Systematic approaches to identify E3 ligase substrates. Biochem. J. **473**, 4083–4101. (10.1042/bcj20160719) 27834739 PMC5103871

[5] Lee JM, Hammarén HM, Savitski MM, Baek SH. 2023 Control of protein stability by post-translational modifications. Nat. Commun. **14**, 1–16. (10.1038/s41467-023-35795-8) 36639369 PMC9839724

[6] Yen HCS, Xu Q, Chou DM, Zhao Z, Elledge SJ. 2008 Global protein stability profiling in mammalian cells. Science **322**, 918–923. (10.1126/science.1160489) 18988847

[7] Fouladkou F, Landry T, Kawabe H, Neeb A, Lu C, Brose N, Stambolic V, Rotin D. 2008 The ubiquitin ligase Nedd4-1 is dispensable for the regulation of PTEN stability and localization. Proc. Natl Acad. Sci. USA **105**, 8585–8590. (10.1073/pnas.0803233105) 18562292 PMC2438405

[8] Kleiger G, Mayor T. 2014 Perilous journey: a tour of the ubiquitin–proteasome system. Trends Cell Biol. **24**, 352–359. (10.1016/j.tcb.2013.12.003) 24457024 PMC4037451

[9] Pla-Prats C, Thomä NH. 2022 Quality control of protein complex assembly by the ubiquitin–proteasome system. Trends Cell Biol. **32**, 696–706. (10.1016/j.tcb.2022.02.005) 35300891

[10] Kuang E, Qi J, Ronai Z. 2013 Emerging roles of E3 ubiquitin ligases in autophagy. Trends Biochem. Sci. **38**, 453–460. (10.1016/j.tibs.2013.06.008) 23870665 PMC3771342

[11] Melino G, Cecconi F, Pelicci PG, Mak TW, Bernassola F. 2019 Emerging roles of HECT‐type E3 ubiquitin ligases in autophagy regulation. Mol. Oncol. **13**, 2033–2048. (10.1002/1878-0261.12567) 31441992 PMC6763782

[12] Lin Q *et al*. 2017 The HECT E3 ubiquitin ligase NEDD4 interacts with and ubiquitinates SQSTM1 for inclusion body autophagy. J. Cell Sci. **130**, 3839–3850. (10.1242/jcs.207068) 29021346

[13] Lee EW, Lee MS, Camus S, Ghim J, Yang MR, Oh W, Ha NC, Lane DP, Song J. 2009 Differential regulation of p53 and p21 by MKRN1 E3 ligase controls cell cycle arrest and apoptosis. EMBO J. **28**, 2100–2113. (10.1038/emboj.2009.164) 19536131 PMC2718286

[14] Chinnam M, Xu C, Lama R, Zhang X, Cedeno CD, Wang Y, Stablewski AB, Goodrich DW, Wang X. 2022 MDM2 E3 ligase activity is essential for p53 regulation and cell cycle integrity. PLoS Genet. **18**, e1010171. (10.1371/journal.pgen.1010171) 35588102 PMC9119546

[15] Dang F, Nie L, Wei W. 2020 Ubiquitin signaling in cell cycle control and tumorigenesis. Cell Death Differ. **28**, 427–438. (10.1038/s41418-020-00648-0) 33130827 PMC7862229

[16] Schaefer A, Nethe M, Hordijk PL. 2012 Ubiquitin links to cytoskeletal dynamics, cell adhesion and migration. Biochem. J. **442**, 13–25. (10.1042/bj20111815) 22280013

[17] Kenneth NS, Duckett CS. 2012 IAP proteins: regulators of cell migration and development. Curr. Opin. Cell Biol. **24**, 871–875. (10.1016/j.ceb.2012.11.004) 23219152

[18] Roberts JZ, Crawford N, Longley DB. 2022 The role of ubiquitination in apoptosis and necroptosis. Cell Death Differ. **29**, 272–284. (10.1038/s41418-021-00922-9) 34912054 PMC8817035

[19] Cruz Walma DA, Chen Z, Bullock AN, Yamada KM. 2022 Ubiquitin ligases: guardians of mammalian development. Nat. Rev. Mol. Cell Biol. **23**, 350–367. (10.1038/s41580-021-00448-5) 35079164

[20] Reyes RV, Hino K, Canales CP, Dickson EJ, La Torre A, Simó S. 2022 The E3 ubiquitin ligase CRL5 regulates dentate gyrus morphogenesis, adult neurogenesis, and animal behavior. Front. Neurosci. **16**, 908719. (10.3389/fnins.2022.908719) 35801174 PMC9253586

[21] Tuoc TC, Stoykova A. 2010 Roles of the ubiquitin-proteosome system in neurogenesis. Cell Cycle **9**, 3194–3200. (10.4161/cc.9.16.12551) 20852390

[22] Stegmüller J, Bonni A. 2010 Destroy to create: E3 ubiquitin ligases in neurogenesis. F1000 Biol. Rep. **2**, 38. (10.3410/B2-38) 20948796 PMC2950039

[23] Kawabe H *et al*. 2010 Regulation of Rap2A by the ubiquitin ligase Nedd4-1 controls neurite development. Neuron **65**, 358–372. (10.1016/j.neuron.2010.01.007) 20159449 PMC2825371

[24] Kawabe H, Brose N. 2011 The role of ubiquitylation in nerve cell development. Nat. Rev. Neurosci. **12**, 251–268. (10.1038/nrn3009) 21505515

[25] Ambrozkiewicz MC *et al*. 2018 Polarity acquisition in cortical neurons is driven by synergistic action of Sox9-regulated Wwp1 and Wwp2 E3 ubiquitin ligases and intronic miR-140. Neuron **100**, 1097–1115.(10.1016/j.neuron.2018.10.008) 30392800

[26] Hamilton AM, Zito K. 2013 Breaking it down: the ubiquitin proteasome system in neuronal morphogenesis. Neural Plast. **2013**, 1–10. (10.1155/2013/196848) PMC3586504 23476809

[27] Song J, Merrill RA, Usachev AY, Strack S. 2021 The X-linked intellectual disability gene product and E3 ubiquitin ligase KLHL15 degrades doublecortin proteins to constrain neuronal dendritogenesis. J. Biol. Chem. **296**, 100082. (10.1074/jbc.RA120.016210) 33199366 PMC7948412

[28] Yamada T, Yang Y, Bonni A. 2013 Spatial organization of ubiquitin ligase pathways orchestrates neuronal connectivity. Trends Neurosci. **36**, 218–226. (10.1016/j.tins.2012.12.004) 23332798 PMC3622823

[29] Yang Y, Kim AH, Bonni A. 2010 The dynamic ubiquitin ligase duo: Cdh1-APC and Cdc20-APC regulate neuronal morphogenesis and connectivity. Curr. Opin. Neurobiol. **20**, 92–99. (10.1016/j.conb.2009.12.004) 20060286 PMC3118470

[30] Ambrozkiewicz MC, Kawabe H. 2015 HECT‐type E3 ubiquitin ligases in nerve cell development and synapse physiology. FEBS Lett. **589**, 1635–1643. (10.1016/j.febslet.2015.05.009) 25979171

[31] Kawabe H, Stegmüller J. 2021 The role of E3 ubiquitin ligases in synapse function in the healthy and diseased brain. Mol. Cell. Neurosci. **112**, 103602. (10.1016/j.mcn.2021.103602) 33581237

[32] Sharma G, Banerjee S. 2022 Activity-regulated E3 ubiquitin ligase TRIM47 modulates excitatory synapse development. Front. Mol. Neurosci. **15**, 943980. (10.3389/fnmol.2022.943980) 36211980 PMC9532517

[33] Ding M, Chao D, Wang G, Shen K. 2007 Spatial regulation of an E3 ubiquitin ligase directs selective synapse elimination. Science **317**, 947–951. (10.1126/science.1145727) 17626846

[34] Furusawa K, Ishii K, Tsuji M, Tokumitsu N, Hasegawa E, Emoto K. 2023 Presynaptic Ube3a E3 ligase promotes synapse elimination through down-regulation of BMP signaling. Science **381**, 1197–1205. (10.1126/science.ade8978) 37708280

[35] Hegde AN. 2010 The ubiquitin-proteasome pathway and synaptic plasticity. Learn. Mem. **17**, 314–327. (10.1101/lm.1504010) 20566674 PMC2904103

[36] Srinivasan B, Samaddar S, Mylavarapu SVS, Clement JP, Banerjee S. 2021 Homeostatic scaling is driven by a translation-dependent degradation axis that recruits miRISC remodeling. PLoS Biol. **19**, e3001432. (10.1371/journal.pbio.3001432) 34813590 PMC8610276

[37] Mabb AM, Ehlers MD. 2018 Arc ubiquitination in synaptic plasticity. Semin. Cell Dev. Biol. **77**, 10–16. (10.1016/j.semcdb.2017.09.009) 28890418

[38] Khatri N, Man HY. 2019 The autism and angelman syndrome protein Ube3A/E6AP: the gene, E3 ligase ubiquitination targets and neurobiological functions. Front. Mol. Neurosci. **12**, 448792. (10.3389/fnmol.2019.00109) PMC6502993 31114479

[39] Kasherman MA, Premarathne S, Burne THJ, Wood SA, Piper M. 2020 The ubiquitin system: a regulatory hub for intellectual disability and autism spectrum disorder. Mol. Neurobiol. **57**, 2179–2193. (10.1007/s12035-020-01881-x) 31974941

[40] Lopez SJ, Segal DJ, LaSalle JM. 2019 UBE3A: an E3 ubiquitin ligase with genome-wide impact in neurodevelopmental disease. Front. Mol. Neurosci. **11**, 425682. (10.3389/fnmol.2018.00476) PMC6338038 30686997

[41] Hegde AN, Upadhya SC. 2007 The ubiquitin–proteasome pathway in health and disease of the nervous system. Trends Neurosci. **30**, 587–595. (10.1016/j.tins.2007.08.005) 17950927

[42] George AJ, Hoffiz YC, Charles AJ, Zhu Y, Mabb AM. 2018 A comprehensive atlas of E3 ubiquitin ligase mutations in neurological disorders. Front. Genet. **9**, 308113. (10.3389/fgene.2018.00029) PMC5817383 29491882

[43] Potjewyd FM, Axtman AD. 2021 Exploration of aberrant E3 ligases implicated in Alzheimer’s disease and development of chemical tools to modulate their function. Front. Cell. Neurosci. **15**, 768655. (10.3389/fncel.2021.768655) 34867205 PMC8637409

[44] Olabarria M, Pasini S, Corona C, Robador P, Song C, Patel H, Lefort R. 2019 Dysfunction of the ubiquitin ligase E3A Ube3A/E6-AP contributes to synaptic pathology in Alzheimer’s disease. Commun. Biol. **2**, 1–14. (10.1038/s42003-019-0350-5) 30937395 PMC6430817

[45] Hildebrandt A, Alanis-Lobato G, Voigt A, Zarnack K, Andrade-Navarro MA, Beli P, König J. 2017 Interaction profiling of RNA-binding ubiquitin ligases reveals a link between posttranscriptional regulation and the ubiquitin system. Sci. Rep. **7**, 1–14. (10.1038/s41598-017-16695-6) 29185492 PMC5707401

[46] Williams FP, Haubrich K, Perez-Borrajero C, Hennig J. 2019 Emerging RNA-binding roles in the TRIM family of ubiquitin ligases. Biol. Chem. **400**, 1443–1464. (10.1515/hsz-2019-0158) 31120853

[47] Zhang Q, Fan L, Hou F, Dong A, Wang YX, Tong Y. 2015 New insights into the RNA-binding and E3 ubiquitin ligase activities of roquins. Sci. Rep. **5**, 1–13. (10.1038/srep15660) PMC4614863 26489670

[48] Cano F, Miranda-Saavedra D, Lehner PJ. 2010 RNA-binding E3 ubiquitin ligases: novel players in nucleic acid regulation. Biochem. Soc. Trans. **38**, 1621–1626. (10.1042/bst0381621) 21118137

[49] Goyani S, Roy M, Singh R. 2021 TRIM-NHL as RNA binding ubiquitin E3 ligase (RBUL): implication in development and disease pathogenesis. Biochim. Biophys. Acta Mol. Basis Dis. **1867**, 166066. (10.1016/j.bbadis.2020.166066) 33418035

[50] Pereira B, Le Borgne M, Chartier NT, Billaud M, Almeida R. 2013 MEX-3 proteins: recent insights on novel post-transcriptional regulators. Trends Biochem. Sci. **38**, 477–479. (10.1016/j.tibs.2013.08.004) 23999169

[51] Buchet-Poyau K, Courchet J, Le Hir H, Séraphin B, Scoazec JY, Duret L, Domon-Dell C, Freund JN, Billaud M. 2007 Identification and characterization of human Mex-3 proteins, a novel family of evolutionarily conserved RNA-binding proteins differentially localized to processing bodies. Nucleic Acids Res. **35**, 1289–1300. (10.1093/nar/gkm016) 17267406 PMC1851655

[52] Conaco C, Otto S, Han JJ, Mandel G. 2006 Reciprocal actions of REST and a microRNA promote neuronal identity. Proc. Natl Acad. Sci. USA **103**, 2422–2427. (10.1073/pnas.0511041103) 16461918 PMC1413753

[53] Ballas N, Grunseich C, Lu DD, Speh JC, Mandel G. 2005 REST and its corepressors mediate plasticity of neuronal gene chromatin throughout neurogenesis. Cell **121**, 645–657. (10.1016/j.cell.2005.03.013) 15907476

[54] Hentze MW, Castello A, Schwarzl T, Preiss T. 2018 A brave new world of RNA-binding proteins. Nat. Rev. Mol. Cell Biol. **19**, 327–341. (10.1038/nrm.2017.130) 29339797

[55] Takada H, Kawana T, Ito Y, Kikuno RF, Mamada H, Araki T, Koga H, Asashima M, Taira M. 2009 The RNA-binding protein Mex3b has a fine-tuning system for mRNA regulation in early Xenopus development. Development **136**, 2413–2422. (10.1242/dev.029165) 19542354

[56] Mandel G, Fiondella CG, Covey MV, Lu DD, LoTurco JJ, Ballas N. 2011 Repressor element 1 silencing transcription factor (REST) controls radial migration and temporal neuronal specification during neocortical development. Proc. Natl Acad. Sci. USA **108**, 16789–16794. (10.1073/pnas.1113486108) 21921234 PMC3189062

[57] Yoon JH *et al*. 2015 Scaffold function of long non-coding RNA HOTAIR in protein ubiquitination. Nat. Commun. **4**, 1–26. (10.1038/ncomms3939) PMC4556280 24326307

[58] Rinn JL *et al*. 2007 Functional demarcation of active and silent chromatin domains in human HOX loci by noncoding RNAs. Cell **129**, 1311–1323. (10.1016/j.cell.2007.05.022) 17604720 PMC2084369

[59] Wang J, Zhao J, Hu P, Gao L, Tian S, He Z. 2022 Long non-coding RNA HOTAIR in central nervous system disorders: new insights in pathogenesis, diagnosis, and therapeutic potential. Front. Mol. Neurosci. **15**, 949095. (10.3389/fnmol.2022.949095) 35813070 PMC9259972

[60] Jin ZL, Gao WY, Liao SJ, Yu T, Shi Q, Yu SZ, Cai YF. 2021 Paeonol inhibits the progression of intracerebral haemorrhage by mediating the HOTAIR/UPF1/ACSL4 axis. ASN Neuro **13**, 175909142110106. (10.1177/17590914211010647) PMC8718120 33906483

[61] Momtazmanesh S, Rezaei N. 2021 Long non-coding RNAs in diagnosis, treatment, prognosis, and progression of glioma: a state-of-the-art review. Front. Oncol. **11**, 712786. (10.3389/fonc.2021.712786) 34322395 PMC8311560

[62] Rouillard AD, Gundersen GW, Fernandez NF, Wang Z, Monteiro CD, McDermott MG, Ma’ayan A. 2016 The harmonizome: a collection of processed datasets gathered to serve and mine knowledge about genes and proteins. Database **2016**, baw100. (10.1093/database/baw100) 27374120 PMC4930834

[63] Tsai MC, Manor O, Wan Y, Mosammaparast N, Wang JK, Lan F, Shi Y, Segal E, Chang HY. 2010 Long noncoding RNA as modular scaffold of histone modification complexes. Science **329**, 689–693. (10.1126/science.1192002) 20616235 PMC2967777

[64] Salamon I, Rasin MR. 2022 Evolution of the neocortex through RNA-binding proteins and post-transcriptional regulation. Front. Neurosci. **15**, 803107. (10.3389/fnins.2021.803107) 35082597 PMC8784817

[65] Draper BW, Mello CC, Bowerman B, Hardin J, Priess JR. 1996 MEX-3 is a KH domain protein that regulates blastomere identity in early C. elegans embryos. Cell **87**, 205–216. (10.1016/s0092-8674(00)81339-2) 8861905

[66] Le Borgne M *et al*. 2014 The RNA-binding protein Mex3b regulates the spatial organization of the Rap1 pathway. Development **141**, 2096–2107. (10.1242/dev.108514) 24803656

[67] Bufalieri F *et al*. 2020 The RNA-binding ubiquitin ligase MEX3A affects glioblastoma tumorigenesis by inducing ubiquitylation and degradation of RIG-I. Cancers **12**, 321. (10.3390/cancers12020321) 32019099 PMC7072305

[68] Baumgart M *et al*. 2014 RNA-seq of the aging brain in the short-lived fish N. furzeri—conserved pathways and novel genes associated with neurogenesis. Aging Cell **13**, 965–974. (10.1111/acel.12257) 25059688 PMC4326923

[69] Yue F *et al*. 2014 A comparative encyclopedia of DNA elements in the mouse genome. Nature **515**, 355–364. (10.1038/nature13992) 25409824 PMC4266106

[70] Samson ML. 1998 Evidence for 3 untranslated region-dependent autoregulation of the Drosophila gene encoding the neuronal nuclear RNA-binding protein ELAV. Genetics **150**, 723–733. (10.1093/genetics/150.2.723) 9755203 PMC1460370

[71] Borgeson CD, Samson ML. 2005 Shared RNA-binding sites for interacting members of the Drosophila ELAV family of neuronal proteins. Nucleic Acids Res. **33**, 6372–6383. (10.1093/nar/gki942) 16282587 PMC1283526

[72] Abe R, Yamamoto K, Sakamoto H. 1996 Target specificity of neuronal RNA-binding protein, Mel-N1: direct binding to the 3’ untranslated region of its own mRNA. Nucleic Acids Res. **24**, 2011–2016. (10.1093/nar/24.11.2011) 8668530 PMC145919

[73] Brooks SA, Connolly JE, Rigby WFC. 2004 The role of mRNA turnover in the regulation of tristetraprolin expression: evidence for an extracellular signal-regulated kinase-specific, AU-rich element-dependent, autoregulatory pathway. J. Immunol. **172**, 7263–7271. (10.4049/jimmunol.172.12.7263) 15187101

[74] Dunlop SA, Tee LBG, Lund RD, Beazley LD. 1997 Development of primary visual projections occurs entirely postnatally in the fat-tailed dunnart, a marsupial mouse, Sminthopsis crassicaudata. J. Comp. Neurol. **384**, 26–40. (10.1002/(sici)1096-9861(19970721)384:13.3.co;2-p) 9214538

[75] Chun J, Shatz C. 1989 The earliest-generated neurons of the cat cerebral cortex: characterization by MAP2 and neurotransmitter immunohistochemistry during fetal life. J. Neurosci. **9**, 1648–1667. (10.1523/jneurosci.09-05-01648.1989) 2566660 PMC6569841

[76] Sarnat HB, Nochlin D, Born DE. 1998 Neuronal nuclear antigen (NeuN): a marker of neuronal maturation in the early human fetal nervous system. Brain Dev. **20**, 88–94. (10.1016/s0387-7604(97)00111-3) 9545178

[77] Xue M *et al*. 2018 HOTAIR induces the ubiquitination of Runx3 by interacting with Mex3b and enhances the invasion of gastric cancer cells. Gastric Cancer **21**, 756–764. (10.1007/s10120-018-0801-6) 29417297

[78] Carbon S, Mungall C. 2024 Gene Ontology data archive. Zenodo https://zenodo.org/records/10536401.

[79] VIB/UGent. 2025 Calculate and draw custom Venn diagrams. See https://bioinformatics.psb.ugent.be/webtools/Venn.

[80] Banerjee S, Neveu P, Kosik KS. 2009 A coordinated local translational control point at the synapse involving relief from silencing and MOV10 degradation. Neuron **64**, 871–884. (10.1016/j.neuron.2009.11.023) 20064393

[81] Lunde A, Glover JC. 2020 A versatile toolbox for semi-automatic cell-by-cell object-based colocalization analysis. Sci. Rep. **10**, 1–26. (10.1038/s41598-020-75835-7) 33149236 PMC7643144

[82] Boulan B, Beghin A, Ravanello C, Deloulme JC, Gory-Fauré S, Andrieux A, Brocard J, Denarier E. 2020 AutoNeuriteJ: an ImageJ plugin for measurement and classification of neuritic extensions. PLoS One **15**, e0234529. (10.1371/journal.pone.0234529) 32673338 PMC7365462

[83] Schmitz SK *et al*. 2011 Automated analysis of neuronal morphology, synapse number and synaptic recruitment. J. Neurosci. Methods **195**, 185–193. (10.1016/j.jneumeth.2011.01.028) 21167201

[84] Peritz T, Zeng F, Kannanayakal TJ, Kilk K, Eiríksdóttir E, Langel U, Eberwine J. 2006 Immunoprecipitation of mRNA-protein complexes. Nat. Protoc. **1**, 577–580. (10.1038/nprot.2006.82) 17406284

[85] Garg K, Sharma G, Samaddar S, Banerjee S. 2025 Supplemetary material from: The E3 ligase MEX3B forms a tripartite complex with Rest and Hotair to determine the proliferative capacity of neural progenitor cells. Figshare. (10.6084/m9.figshare.29086646)40925537

[86] Garg K, Sharma G, Samaddar S, Banerjee S. 2025 Supplementary material from: The E3 ligase MEX3B forms a tripartite complex with Rest and Hotair to determine the proliferative capacity of neural progenitor cells. Figshare. (10.6084/m9.figshare.c.7979995)40925537

